# Tumor necrosis factor alpha antagonism improves neurological recovery in murine intracerebral hemorrhage

**DOI:** 10.1186/1742-2094-10-103

**Published:** 2013-08-20

**Authors:** Beilei Lei, Hana N Dawson, Briana Roulhac-Wilson, Haichen Wang, Daniel T Laskowitz, Michael L James

**Affiliations:** 1 Multidisciplinary Neuroprotection Laboratories, 132 Sands Bldg, Durham, NC 27710, USA; 2 Department of Anesthesiology, DUMC - 3094, Durham, NC 27710, USA; 3 Department of Neurology, DUMC - 2900, Durham, NC 27710, USA; 4 Department of Neurobiology, DUMC - 3209, Durham, NC 27710, USA

**Keywords:** Intracerebral hemorrhage, Microglia, Tumor necrosis factor alpha antagonism, Murine model, Cytokine, Remicade

## Abstract

**Background:**

Intracerebral hemorrhage (ICH) is a devastating stroke subtype characterized by a prominent neuroinflammatory response. Antagonism of pro-inflammatory cytokines by specific antibodies represents a compelling therapeutic strategy to improve neurological outcome in patients after ICH. To test this hypothesis, the tumor necrosis factor alpha (TNF-α) antibody CNTO5048 was administered to mice after ICH induction, and histological and functional endpoints were assessed.

**Methods:**

Using 10 to 12-week-old C57BL/6J male mice, ICH was induced by collagenase injection into the left basal ganglia. Brain TNF-α concentration, microglia activation/macrophage recruitment, hematoma volume, cerebral edema, and rotorod latency were assessed in mice treated with the TNF-α antibody, CNTO5048, or vehicle.

**Results:**

After ICH induction, mice treated with CNTO5048 demonstrated reduction in microglial activation/macrophage recruitment compared to vehicle-treated animals, as assessed by unbiased stereology (*P* = 0.049). This reduction in F4/80-positive cells was associated with a reduction in cleaved caspase-3 (*P* = 0.046) and cerebral edema (*P* = 0.026) despite similar hematoma volumes, when compared to mice treated with vehicle control. Treatment with CNTO5048 after ICH induction was associated with a reduction in functional deficit when compared to mice treated with vehicle control, as assessed by rotorod latencies (*P* = 0.024).

**Conclusions:**

Post-injury treatment with the TNF-α antibody CNTO5048 results in less neuroinflammation and improved functional outcomes in a murine model of ICH.

## Introduction

Intracerebral hemorrhage (ICH) is a devastating form of cerebrovascular disease with higher morbidity and mortality than ischemic stroke [1] but without any proven therapy beyond supportive care. Much of the current clinical research focuses on hematoma reduction and resolution [2,3], as hemorrhage evolution and volume are highly correlated with outcome after ICH [4]. However, the failure of recent large clinical trials [5,6] may be the result of an inability to fully account for the influence of secondary injury from the neuroinflammatory response. Emerging evidence suggests that this inflammatory response may be tied to outcome and may be modifiable [7,8]. Future therapeutic strategies may be aimed at reducing neuroinflammation and inducing or enhancing recovery mechanisms.

Microglia are the resident immune cells in the central nervous system (CNS). Microglial activation and macrophage infiltration remain the cornerstone of the brain’s acute response to injury. Microglia are activated through a variety of different mechanisms and have both destructive and adaptive roles after ICH [9]. Preclinical evidence suggests that microglial modulation might improve recovery after ICH [10]. Secretion of inflammatory mediators, such as tumor necrosis factor alpha (TNF-α), is a major microglial responses to activation, and modulation of this response may be a promising therapeutic strategy.

TNF-α antagonism has been demonstrated as effective therapy for a number of chronic inflammatory states [11]. Further, TNF-α modulation may represent a link between chronic and acute neuroinflammatory states. To test the hypothesis that TNF-α antagonism is a promising therapeutic strategy in acute neuroinflammatory states, a murine analog of the human monoclonal antibody infliximab, CNTO5048, was tested in a murine model of ICH.

## Methods

This specific research project and all animal procedures involved were designed to minimize animal discomfort and numbers, conform to international guidelines on the use of animals, and were approved by the Duke University Institutional Animal Care and Use Committee.

### Experimental groups

Male C57BL/6J mice (10 to 12 weeks of age; Jackson Laboratory, Bar Harbor, ME, USA) were used in these experiments. Prior to ICH induction, mice were randomly assigned to receive either vehicle (phosphate-buffered saline (PBS)) or CNTO5048. Vehicle control (PBS) was used in all experiments.

After randomization, ICH was induced by an operator blinded to treatment group (BL). To assess the translational potential of CNTO5048, a separate observer (HW) blinded to treatment group administered 100 μL of either PBS or CNTO5048 via tail vein injection at 30 minutes after injury. These time points for administration and outcome measurements were chosen based on prior work with promising therapeutics and neuroinflammatory metrics [7,10,12-14]. Sham animals were not included in these experiments as prior work demonstrates that they behave like uninjured animals [7,10,13,14]. Separate observers (HND, BL, BRW), blinded to treatment group, assessed five separate cohorts of mice as follows:

**Figure 1:**
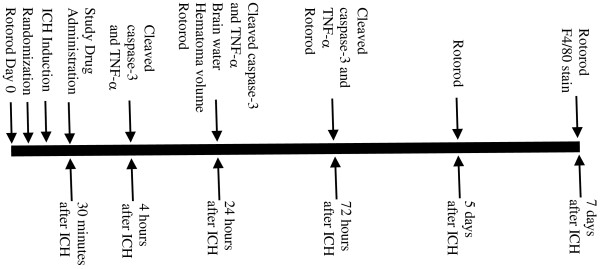
**Timeline of experiments.** Separate cohorts of animals were used to assess brain TNF-α and cleaved caspase-3 levels, F4/80 cell positivity, brain water content, hematoma volume, and rotorod latencies.

Cohort 1 - Regulation of brain TNF-α. At 4, 24, and 72 hours after ICH induction, the expressions of TNF-α and cleaved caspase-3 protein were measured in the ipsilateral hemisphere, using enzyme-linked immunosorbent assay, bicinchoninic acid assay, and western blot analysis, respectively (n = 3 mice/group). The same brain homogenates were used for enzyme-linked immunosorbent assay and western blot analysis.

Cohort 2 - Unbiased microglial/macrophage quantification. At 7 days after ICH induction, immunohistochemical F4/80 staining and cell counting of bilateral hippocampi was performed (n = 6 mice/group). Hippocampi were used based on their relationship to learning and memory, long-term neurological outcome measures, and our own experience [7,10,12-14].

Cohort 3 - Hematoma volume. At 24 hours after ICH induction, hematoma volume was measured by volumetric measurement of hematoxylin and eosin lesionstaining (n = 7 mice/group).

Cohort 4 - Cerebral edema. At 24 hours after ICH induction, cerebral edema was assessed by 'wet-to-dry' brain water content measurement (n = 5 mice/group).

Cohort 5 - Vestibulomotor testing. On Days 0, 1, 3, 5, and 7 after ICH induction, vestibulomotor function was assessed by comparing within animal rotorod (RR) latencies before and after injury (n = 17 to 18 mice/group). Finally, mortality was compared between treatment groups in mice surviving 24 hours or longer after injury.

A timeline of the experiments is provided in Figure 1. Time points for the assessments were chosen based on prior experience with this model [7,10,13,14].

### Anesthetic technique

Mice were anesthetized with 4.6% isoflurane in 30% O_2_/70% N_2_. After anesthetic induction and loss of response to tail stimulation, the trachea was intubated, and the lungs were mechanically ventilated with 1.6% isoflurane in 30% O_2_/70% N_2_. Rectal temperature was maintained at 37.0°C ± 0.2°C by an underbody circulating waterbed.

### Intracerebral hemorrhage model

The mouse ICH model has been reported previously [13]. After anesthetic induction and intubation, the animal’s head was secured in a stereotactic frame. The scalp was incised and a burr hole was created 2.2 mm left lateral to bregma. A 1.0-μL syringe (Hamilton, Reno, NV, USA) with a 25-gauge needle was mounted on the stereotactic frame. The eye of the needle was advanced to a depth of 3 mm from the cortical surface. Type IV-S clostridial collagenase (Sigma, St. Louis, MO, USA; 0.075 U in 0.4 μL 0.9% NaCl) was injected over 2 minutes, and the needle was held motionless for an additional 5 minutes. After slowly withdrawing the needle, the incision was closed, and animals were allowed to recover spontaneous ventilation with subsequent extubation. Following recovery in a warm, non-stimulating environment, mice were allowed free access to food and water.

### Preparation and administration of CNTO5048

CNTO5048, an anti-mouse TNF-α antibody (6.73 mg/mL in 1X PBS stock) was kindly provided by Janssen Biotech (Horsham, PA, USA). For all experiments, CNTO5048 was diluted in PBS at 1.75 mg/mL prior to use. A dose of 7 mg/kg was used for all experiments. This dose was based on the FDA-approved titration of infliximab from 5 to 10 mg/kg for chronic inflammatory diseases [15].

### Quantification of tumor necrosis factor alpha

After anesthetic induction, mice were perfused transcardially with 30 mL PBS. Brains were dissected and flash frozen in liquid nitrogen, and then stored at -80°C. Pulverized injured hemispheres were sonicated on ice in homogenization buffer (20 mM Tris-HCl, pH 8.0, 137 mM NaCl, 2 mM EDTA, 10% glycerol, 1% Triton X-100, and complete protease inhibitor cocktail tablets; Roche Diagnostics, Mannheim, Germany) for 20 seconds. Brain homogenates were incubated on ice for 20 minutes, and then spun at 10,000 X g at 4°C for 10 minutes. The supernatant was removed, aliquotted, and stored at -80°C for protein analysis. Total protein concentrations were measured using a bicinchoninic acid protein assay reagent kit (Pierce Biotechnology, Rockford, IL, USA). TNF-α concentrations in whole-brain homogenates were determined using an enzyme-linked immunosorbent assay kit for mouse TNF-α according to the manufacturer’s instructions (Invitrogen, Camarillo, CA, USA).

### Quantification of cleaved caspase-3

From the isolated supernatant isolated, equal amounts of protein samples were electrophoresed on 4% to 20% SDS-polyacrylamide gels, and transferred to polyvinylidene difluoride membranes (Bio-Rad, Hercules, CA, USA). The membranes were blocked in 5% milk and incubated with cleaved caspase-3 (Asp175) antibody (1:500; Cell Signaling Technology, Beverly, MA, USA) overnight at 4°C. After incubation with secondary horseradish peroxidase-conjugated goat anti-rabbit IgG antibody (Pierce Biotechnology), the blots were detected using Western Dura Extended Duration Substrate (Pierce Biotechnology). Membranes were stripped of immunoglobulin and re-probed using anti-mouse glyceraldehyde-3-phosphate dehydrogenase (GAPDH, 1:2,000; Cell Signaling).

### Immunohistochemistry and stereological analysis

After anesthetic induction, mice were subjected to transcardial perfusion with 30 mL PBS. The brains were rapidly removed and immersion-fixed in 4% formaldehyde for 24 hours, then transferred into 30% sucrose/1x PBS, and stored at 4°C for 48 hours. Frozen coronal sections (40 μm) were collected on a freezing sliding microtome. Floating brain sections were incubated in 1% hydrogen peroxide, permeabilized by 0.1% Saponin, and blocked with 10% goat serum. Sections were incubated overnight with monoclonal rat anti-mouse F4/80 antibody specific for activated microglia and macrophage (1:20,000; Serotec, Raleigh, NC, USA), and biotinylated goat anti-rat immunoglobulin G secondary antibody (1:3000; Vector Laboratories, Inc., Burlingame, CA, USA) was then applied for 1 hour, followed by avidin-biotin-peroxidase complex treatment for 1 hour (ABC kit; Vector Laboratories, Inc., Burlington, CA, USA). Staining was visualized with diaminobenzidine (DAB kit; Vector Laboratories, Inc.). After being mounted onto slides, all sections were counterstained with hematoxylin (Fisher Scientific, Fair Lawn, NJ, USA). Cells were counted using a Nikon 218912 light microscope interfaced with the StereoInvestigator software package (MicroBrightField, Williston, VT, USA). The number of stained cells per volume of the hippocampus was estimated by using an optical fractionator method as previously described [7].

### Measurement of hematoma volume

After anesthetic induction, mice were decapitated, and the brains were removed, flash frozen in 2-methyl butane (-20°C), and stored at -80°C. Coronal sections 20-μm thick were serially taken at 400-μm intervals over the rostral-caudal extent of the lesion. The sections were stained with hematoxylin and eosin, and lesion area was measured by digitally sampling stained sections with an image analyzer (MCID Elite™, Interfocus Imaging, Linton, England). Hematoma volumes (mm^3^) were computed as running sums of lesion area multiplied by the known interval between sections (400 μm) over the extent of the lesion, and expressed as an orthogonal projection.

### Measurement of brain water content

After anesthetic induction, mice were decapitated. Brains were harvested and sectioned mid-sagittally, and each hemisphere was weighed immediately (‘wet’ weight). Hemispheres were allowed to dehydrate over 24 hours at 105°C, and were then re-weighed (‘dry’ weight). Cerebral edema was expressed as calculated water content ((wet weight - dry weight)/(wet weight) x 100).

### Rotorod testing

An automated RR (Ugo Basile, Comerio, Italy) was used to assess within animal differences in vestibulomotor function [16]. On the day before ICH induction, mice underwent two consecutive conditioning trials at a set rotational speed of 16 revolutions/minute for 60 seconds. These were followed by three additional trials with 15-minute intervals and an accelerating rotational speed. The average time to fall from the rotating cylinder in the final three trials was recorded as baseline latency. After ICH induction, three trials with accelerating rotational speed were conducted on post-injury days 1, 3, 5, and 7. The average latency to fall from the rod was recorded. A latency value of 0 seconds indicated inability to grasp the rotating rod.

### Statistical analysis

One-way ANOVA was used to compare TNF-α protein concentration and cleaved caspase-3 protein band densities among groups. Student’s *t*-test was used to compare hematoma volume, brain water content, and F4/80 cell counts. Repeated-measures analysis of variance (ANOVA) with time as the repeated variable was used to compare RR performance to test group effect as a function of time. Bonferroni correction was used for repeated measures technique in ANOVA. Due to differences in pre-injury latencies, post-injury latencies are reported as percentages of the baseline. A *P* value <0.05 was considered statistically significant. All values were expressed as mean ± SD.

## Results

To assess the effects of TNF-α antagonism on microglial activation, brain TNF-α concentration and downstream protein expression were measured at 4, 24, and 72 hours after ICH since TNF-α is an early product of neutrophil and microglial activation. Brain TNF-α expression was not affected by CNTO5048 administration (CNTO5048 versus vehicle: 1745 ± 649 versus 1352 ± 21 pg/g at 4 hours after ICH, *P* = 0.354; 995 ± 161 versus 805 ± 38 pg/g at 24 hours after ICH, *P* = 0.1179; and 445 ± 58 versus 460 ± 77 pg/g at 72 hours after ICH, *P* = 0.801). Notably, brain TNF-α protein concentration peaked at 4 hours after ICH, and then gradually decreased over time, which is consistent with a previous report [17].

**Figure 2:**
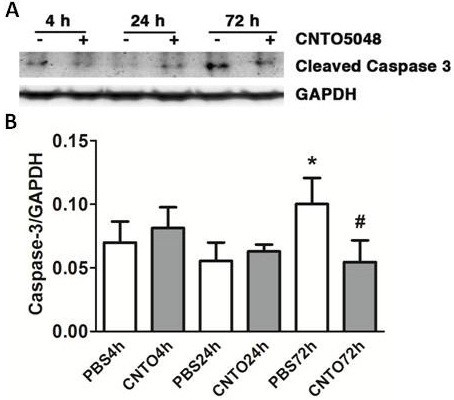
**Cleaved caspase-3 expression after intracerebral hemorrhage.** Representative cleaved caspase-3 western blots **(A)** and band density measurements **(B)** demonstrate reduction at 4, 24, and 72 hours after intrastriatal collagenase injection in mice given 7 mg/kg CNTO5048 or an equivalent volume of phosphate-buffered saline (PBS) via tail vein injection at 30 minutes after injury. (ANOVA; *P = 0.045 compared to PBS at 4 hours, ^#^*P* = 0.046 compared to PBS at 72 hours; n = 3/group) CNTO4h, CNTO5048-treated mice at 4 hours after injury; CNTO24h, CNTO5048-treated mice at 24 hours after injury; CNTO72h, CNTO5048-treated mice at 72 hours after injury; GAPHD, glyceraldehyde-3-phosphate dehydrogenase; h, hours; PBS4h, PBS-treated mice at 4 hours after injury; PBS24h, PBS-treated mice at 24 hours after injury; PBS 72h, PBS-treated mice at 72 hours after injury.

TNF-α activates of the caspase-3 pathway, resulting in neuronal apoptosis. Moreover, it has been reported that apoptotic caspases can activate microglia [18]. Therefore, cleaved caspase-3 (that is, activated caspase-3) was assessed in ipsilateral hemispheres after neutralization of TNF-α by CNTO5048 administration. In the same brain samples used to measure TNF-α concentration, the CNTO5048-treated group demonstrated a reduction in cleaved caspase-3 compared to the vehicle-treated group at 72 hours after ICH (Figure 2).

**Figure 3:**
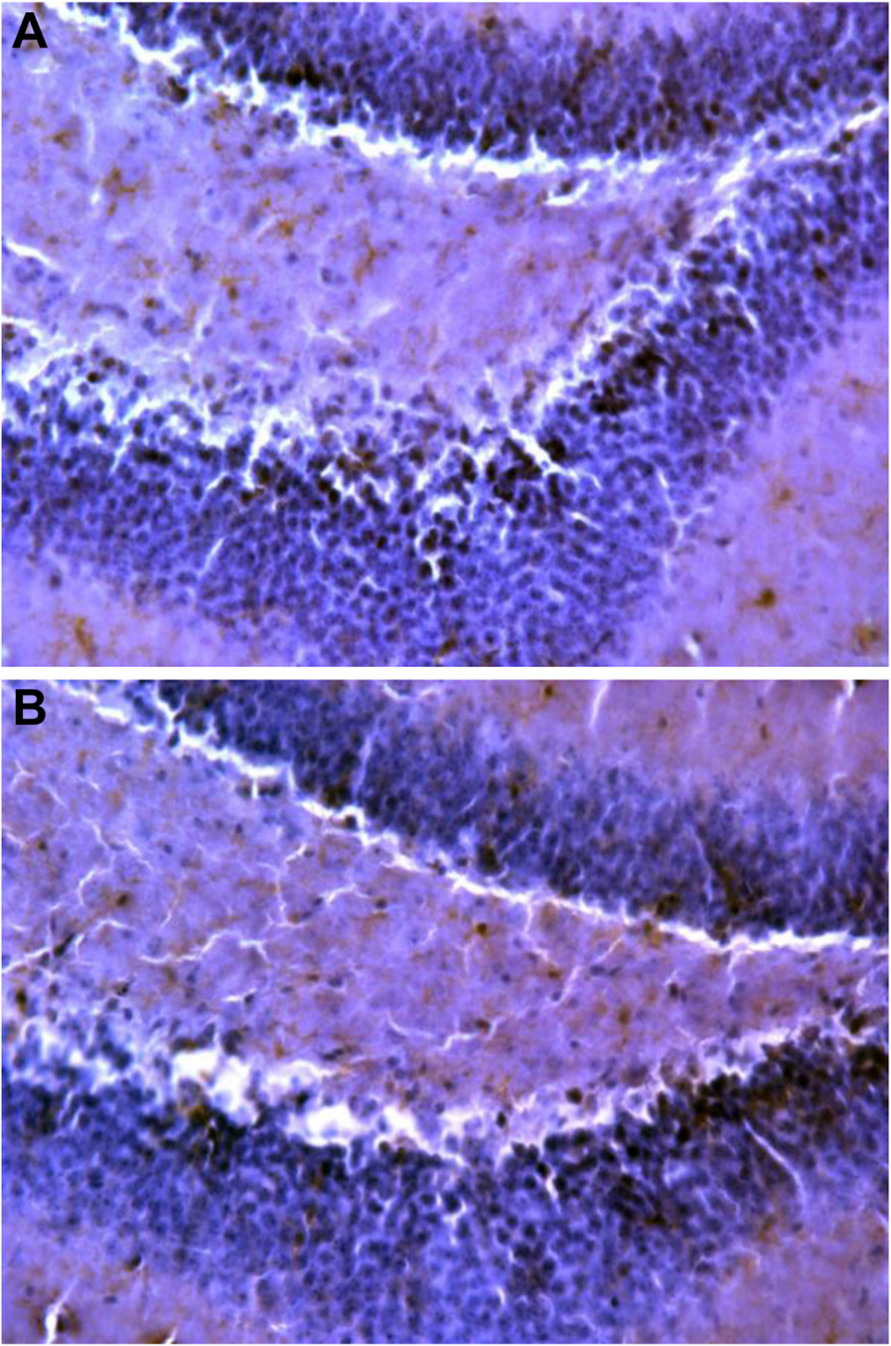
**Macrophage recruitment/microglial activation after intracerebral hemorrhage.** Representative photos of F4/80 positive cells in ipsilateral hippocampus are shown from CNTO5048-treated **(A)** and phosphate-buffered saline-treated **(B)** mice. As a measure of microglial activation/macrophage recruitment, F4/80-positive cells were reduced in the ipsilateral hippocampus 7 days after intrastriatal collagenase injection in mice given 7 mg/kg CNTO5048 compared to those treated with an equivalent volume of phosphate-buffered saline via tail vein injection at 30 minutes after injury. (CNTO5048 versus vehicle: 3,880 ± 949 versus 4,953 ± 691 cells/mm^3^; *t*-test; *P = 0.049; n = 6/group).

In this model of ICH, cerebral edema is associated with the extent of microglial activation and macrophage recruitment, leading to an increase in vascular permeability [10,12,13]. To assess the effects of CNTO5048 on microglial activation/macrophage recruitment, F4/80 staining was performed at 7 days after ICH. The CNTO5048-treated group demonstrated reduction in F4/80-positive cells in the ipsilateral hippocampus compared to the vehicle-treated group (Figure 3).

**Figure 4:**
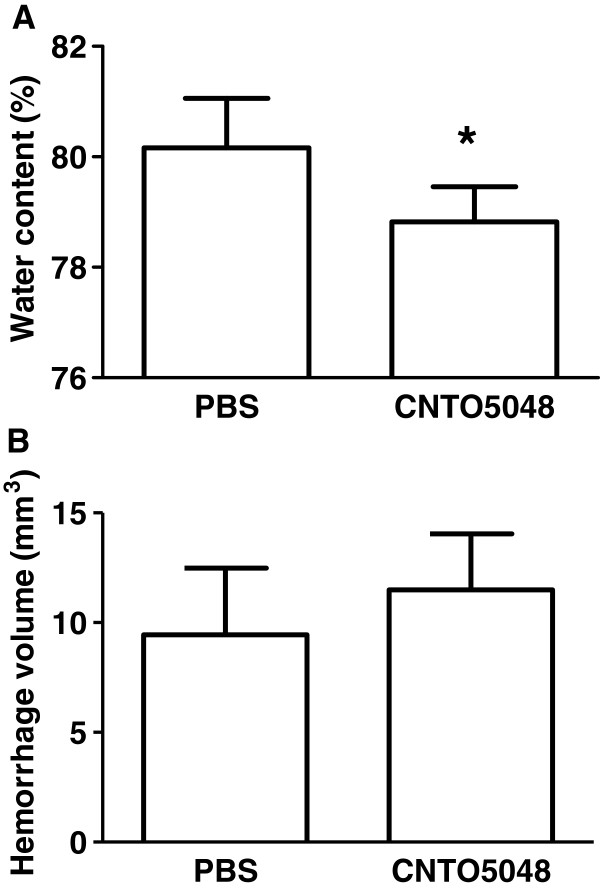
**Brain water content and hemorrhage volume after intracerebral hemorrhage.** Brain water content **(A)** was decreased, while hemorrhage size **(B)** was unaffected at 24 hours after intrastriatal collagenase injection in mice given 7 mg/kg CNTO5048 compared to those treated with an equivalent volume of phosphate-buffered saline via tail vein injection at 30 minutes after injury. (CNTO5048 versus vehicle: 78.82% ± 0.64 versus 80.16% ± 0.89; *t*-test; *P = 0.026; n = 5/group).

Functional recovery after ICH is directly related to hematoma volume and cerebral edema, which is a consequence of neuroinflammation [8,19,20]. By 24 hours after ICH, cerebral edema is maximal, and hemorrhage evolution has stabilized. Thus, the effects of TNF-α antagonism on brain water content and hematoma volume were assessed between the groups at 24 hours after injury. The CNTO5048-treated group demonstrated reduced brain water content compared to the vehicle-treated group at this time point (Figure 4). At the same time, hematoma volume was equivalent between the groups (CNTO5048 versus vehicle: 11.49 ± 2.54 versus 9.44 ± 3.04 mm^3^; *P* = 0.304).

**Figure 5:**
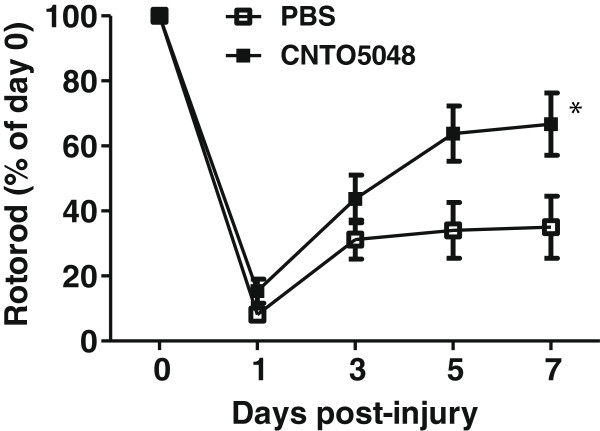
**Rotorod latencies after intracerebral hemorrhage.** Over the first 7 days after intrastriatal collagenase injection, rotorod latencies were longer in mice given 7 mg/kg CNTO5048 compared to those treated with an equivalent volume of phosphate-buffered saline via tail vein injection at 30 minutes after injury. Due to differences in pre-injury latencies, post-injury latencies are reported as percentages of the baseline. Shams are not shown as they behave like uninjured animals, as previously demonstrated. (ANOVA; *P = 0.0243; n = 14/group; error bars represent standard deviation).

Potential clinical efficacy of a novel therapeutic strategy is difficult to assess without demonstration of functional recovery after injury in the preclinical setting. To assess vestibulomotor functional recovery over the acute to subacute phase of injury, the effects of TNF-α antagonism on RR latencies were assessed over the first 7 days after ICH. All animals were able to grasp the rod on all testing days. However, the CNTO5048-treated group demonstrated greater return of RR latencies after injury toward baseline values when compared to the vehicle-treated group over this time period (Figure 5). Post-injury latencies are reported as percentages of the baseline latency due to differences in baseline RR latencies. Finally, the overall mortality was lower in the CNTO5048-treated group than in the vehicle-treated group (13.16% versus 23.08%; *P* = 0.04).

## Discussion

Administration of the TNF-α antibody CNTO5048 decreased mortality and improved short-term recovery of vestibulomotor function while decreasing neuroinflammation in our murine model of ICH. While TNF-α antagonism using monoclonal antibodies is a well-established treatment for rheumatological disease, the present findings are the first to demonstrate its potential efficacy for improving recovery after ICH.

While the blood-brain barrier (BBB) remains impermeable to large molecules for the first 12 hours after ICH [21], permeability increases over the first 24 hours allowing central nervous system (CNS) exposure to previously excluded substances, such as blood-borne cytokines including TNF-α. Systemic and *in situ* cytokines contribute to cerebral edema evolution [22], which then influences functional outcome [19,23,24]. However, the exact mechanism of edema formation and its contribution to ICH-induced neurological deficits needs further investigation [25].

Direct injury to CNS cellular components results in the release of cytokines from immunomodulatory cells such as microglia, ultimately enhancing edema formation [26-29]. TNF-α is singularly important. It is one of the first inflammatory cytokines to be expressed after injury. TNF-α directly upregulates p-selectin on endovascular cells, which then interact with integrins leading to leukocyte infiltration. Preclinical studies [30-32] and human investigations [33,34] have linked increased cytokine concentrations with cerebral edema progression and outcome after ICH.

Though its exact role remains undefined, TNF-α does not serve as a simple ‘biomarker’ of inflammation, but rather plays a central role in mediating and extending neuronal injury after insult [35]. In preclinical models, brain TNF-α expression is upregulated after ICH. Knockout of TNF-α or inhibition of TNF-α by a TNF-α-specific antisense oligodeoxynucleotide is neuroprotective against ICH [17,36]. In CNS microvascular endothelial cells, TNF-α can act through NF-κB signaling to repress claudin-5 promoter activity and therefore downregulate claudin-5 expression. Since claudin-5 expression is important for tight junctions, downregulation may be a potential molecular mechanism of BBB disruption in inflammatory CNS conditions [37]. Lastly, preclinical data suggest that modification of TNF-α expression [10,19], and infliximab in particular [38], attenuates the immune response after ICH, resulting in neuroprotection.

For these reasons, CNTO5048, a murine infliximab analog, was tested for its initial potential for translation as a therapeutic to improve recovery after ICH. Infliximab is currently FDA-approved for treating chronic inflammatory states such as rheumatoid arthritis. In addition, infliximab has demonstrated effects in both hippocampal cell culture [39], and in peripheral nerve injury models where it reduces brain-derived neurotrophic factor [40]. While data for its use in acute CNS injury are limited, especially in ICH, TNF-α antagonism as a therapeutic strategy has gained attention in models of other forms of injury such as ischemic stroke [41,42], traumatic brain injury [43,44], and spinal cord injury [45].

While these data are encouraging, several issues should be addressed before translation to human disease. First, pre-injury RR latency differences are a possible confounding variable. Baseline latency differences might suggest fundamental group differences despite identical genetic background, animal age, testing methods, etcetera. Further, the obvious question is whether CNTO5048 can cross the BBB after injury. As systemic inflammation is known to affect brain inflammation [46], it is possible that the present results are due to systemic TNF-α antagonism; however, reduction in brain cleaved caspase-3 lends evidence to the effects of CNTO5048 in the brain. This is further supported by a lack of effect on brain TNF-α expression, as transcription should not be affected by receptor antagonism. Future studies should assess additional downstream products of the TNF-α receptor in the brain. It would also be of great interest to determine which cell types (that is, glial versus neuronal) might be preferentially bound by the drug, and if the effects of CNTO5048 are brain region-specific (that is, hippocampal versus cortical).

While the present study rigorously adhered to stroke therapy academic industry roundtable criteria [47], future experiments should assess other variables as outlined in the drug development guidelines from the National Institute for Neurological Disorders and Stroke [48], specifically demonstration of the optimal dose and therapeutic window. Further, long-term outcomes were not assessed in these experiments, and future experiments with CNTO5048 should include neurobehavioral assessment at one month or more after injury. In addition, while CNTO5048 was tolerated in this study, the most obvious concern would be an increase in infections that might be associated with cytokine antagonism. True dose-response, therapeutic window, and toxicity studies would discern whether this risk is theoretical or concerning, and would also define important parameters for administration. Future studies should also include female and aged animals, and extrapolation of these findings to higher order animals.

Bearing this in mind, a number of factors are encouraging for rapid translation of infliximab to treat ICH. First, the drug is currently FDA-approved for treatment of inflammatory states. It is easily administered, and human toxicity, as well as drug tolerance, data are available from years of use since its approval. ICH is a disease without proven therapy, and murine models of ICH are high throughput. Further, monoclonal antibodies against TNF-α make sense as a therapeutic strategy in ICH due to the marked neuroinflammatory effects seen in the disease. Thus, in light of the present findings, future study of CNTO5048 might easily address some of these outstanding issues, so that infliximab will readily progress into early-phase human clinical trial in ICH.

## Conclusions

Post-injury administration of the TNF-α monoclonal antibody CNTO5048 improved recovery of vestibulomotor function in a murine model of ICH that was associated with reduced neuroinflammation demonstrated by decreased cerebral edema and cleaved caspase-3. While encouraging for potential translation of this therapeutic strategy, future research should explore the potential mechanism, determine the optimal dose and timing of administration, and assess efficacy in female, aged, and higher order animals.

## Abbreviations

ANOVA: Analysis of variance; BBB: Blood-brain barrier; CNS: Central nervous system; ICH: Intracerebral hemorrhage; FDA: Food and drug administration; GAPDH: Glyceraldehyde-3-phosphate dehydrogenase; PBS: Phosphate-buffered saline; RR: Rotorod; TNF-α: Tumor necrosis factor alpha.

## Competing interests

Michael L. 'Luke' James MD receives grant funding from the American Heart Association, National Institutes of Health, Baxter, Cephalogics, and Hospira. He is also a consultant for Cephalogics and Hospira. All other authors declare that they have no competing interests.

## Authors’ contributions

BL performed experimental design, animal surgery, enzyme-linked immunosorbent assays, western blotting, immunohistochemical analyses, data interpretation, and manuscript preparation. HND performed immunohistochemical analyses, data interpretation, and manuscript preparation. BRW performed testing for vestibulomotor function, immunohistochemical analyses, and manuscript preparation. HW performed experimental design, data interpretation, and manuscript preparation. DTL performed experimental design, data interpretation, and manuscript preparation. MLJ performed experimental design, data interpretation, and manuscript drafting and revision. All authors have read and approved the final version of the manuscript.

## Authors’ information

Dr. Beilei Lei is a research scientist in the Multidisciplinary Neuroprotection Laboratories at Duke Medical Center. With advanced training in both medicine and molecular pharmacology and pathology, Dr. Lei has a solid foundation in the requisite skillset for translational research.

Dr. Hana Dawson’s research interests are focused on the role of tau protein in neurodegenerative disorders involving deposition of abnormal tau protein isoforms in neurons and glial cells in the brain. Using diverse models, her goal is to clarify the role of tau-related neuropathology and to uncover strategies to treat human tauopathies using molecular, biochemical, cellular, and whole animal techniques.

Dr. Haichen Wang is a faculty member in the Multidisciplinary Neuroprotection Laboratories at Duke University, Dr. Wang uses bio-molecular techniques and clinically relevant animal models to study mechanisms and acute treatment of cerebrovascular disorders, and brain and spinal cord injuries.

Dr. Daniel T. Laskowitz is a principal investigator in the Multidisciplinary Neuroprotection Laboratories at Duke University, Dr. Laskowitz uses molecular biology, cell culture, and animal modeling techniques to examine the CNS response to acute injury. In particular, he is interested in the role of microglial activation and the endogenous CNS inflammatory response in exacerbating secondary injury following acute brain insult. Much of the *in vitro* work in this laboratory is dedicated to elucidating cellular responses to injury with the ultimate goal of developing new therapeutic interventions in the clinical setting of stroke, intracranial hemorrhage, and closed-head injury.

Dr. Michael L. 'Luke' James is the associate director and a principal investigator of the Multidisciplinary Neuroprotection Laboratories and the Brain Injury Translational Research Center at Duke University, Dr. James incorporates a truly translational approach to research in acute CNS injury. Specifically interested in microglial modulation after injury to improve patient outcomes, he manages research projects in both the preclinical (MNL) and clinical (BITR) arenas.
